# A Venom Gland Extracellular Chitin-Binding-Like Protein from Pupal Endoparasitoid Wasps, *Pteromalus Puparum*, Selectively Binds Chitin

**DOI:** 10.3390/toxins7124867

**Published:** 2015-11-30

**Authors:** Yu Zhu, Xin-Hai Ye, Yang Liu, Zhi-Chao Yan, David Stanley, Gong-Yin Ye, Qi Fang

**Affiliations:** 1 State Key Laboratory of Rice Biology & Key Laboratory of Agricultural Entomology of Ministry of Agriculture, Institute of Insect Sciences, Zhejiang University, Hangzhou 310058, China; yuzhu@zju.edu.cn (Y.Z.); sammie90@126.com (Y.L.); yan_zc@126.com (Z.-C.Y.); chu@zju.edu.cn (G.-Y.Y.); 2 The College of Agriculture, South China Agricultural University, Guangzhou 510642, China; yexinhai1204@hotmail.com; 3 Biological Control of Insects Research Laboratory, USDA/Agricultural Research Service, Columbia, MO 65203, USA; stanleyd@missouri.edu

**Keywords:** parasitoid, chitin binding protein, venom apparatus, venom proteins

## Abstract

Chitin-binding proteins (CBPs) are present in many species and they act in a variety of biological processes. We analyzed a *Pteromalus puparum* venom apparatus proteome and transcriptome and identified a partial gene encoding a possible CBP. Here, we report cloning a full-length cDNA of a sequence encoding a chitin-binding-like protein (PpCBP) from *P. puparum*, a pupal endoparasitoid of *Pieris rapae*. The cDNA encoded a 96-amino-acid protein, including a secretory signal peptide and a chitin-binding peritrophin-A domain. Phylogenetic analysis of chitin binding domains (CBDs) of cuticle proteins and peritrophic matrix proteins in selected insects revealed that the CBD of PpCBP clustered with the CBD of *Nasonia vitripennis*. The *PpCBP* is specifically expressed in the venom apparatus of *P. puparum*, mostly in the venom gland. *PpCBP* expression was highest at day one after adult eclosion and much lower for the following five days. We produced a recombinant PpCBP and binding assays showed the recombinant protein selectively binds chitin but not cellulose *in vitro*. We infer that PpCBP serves a structural role in the venom reservoir, or may be injected into the host to help wound healing of the host exoskeleton.

## 1. Introduction

Chitin-binding proteins (CBPs) are ubiquitous in organisms from viruses to invertebrates. Depending on the species, CBPs are involved in various biological functions, such as binding to chitin, antimicrobial activities, enhancing chitinolytic activity and hydrophobic surface sensing [1,2,3]. In insects, CBPs are major components of insect cuticles and peritrophic membranes (PMs). PMs act in facilitating digestive processes by compartmentalizing insect guts and protecting against microbial infections [4]. Insect cuticle serves as an exoskeleton, a barrier to reduce water loss, and as a form of physical immunity for protection from infection and invasion [5]. Insects express two CBP classes. PM CBPs usually feature a cysteine-rich chitin binding domain (CBD) including some conserved aromatic amino acids [6]. Cuticular CBPs typically include a Rebers and Riddiford Consensus sequence (R & R Consensus; pfam00379;) that lacks cysteine residues [7]. The common motif of the R & R Consensus was G-X(8)-G-X(6)-Y-X-A-X-E-X-GY-X(7)-P-X-P or a modification of it: G-X(7)-[DEN]-G-X(6)-[FY]-X-A-[DGN]-X(2,3)-G-[FY]-X-[AP]-X(6) (where X represents any amino acid) [8]. Three CBPs were discovered in the potato hornworm hemolymph [9], suggesting that CBPs may have unexpected functions in addition to structural roles in the PM and cuticle.

Most endoparasitoid wasps inject venom into their host hemocoels at oviposition. The venom and various venom-associated compounds suppress host immunity and manipulate host physiology for successful progeny development [10]. Some venom factors may have no functions during oviposition. Alternatively, they may be involved in post-translational modification of other venom proteins, or act in venom gland functions [11]. Venom proteins with sequence motifs associated with chitin-binding have been identified in venom glands. A CBP has been reported in the venom gland of the parasitoid *Nasonia vitripennis* [12]. In the venom of *Chelonus inanitus*, two mucin-like peritrophins, Ci-23c and Ci220, which have chitin-binding peritrophin domains, were identified using expressed sequence tag (EST) sequencing and proteomics [13]. Also, two endocuticular venom proteins with R & R consensus sequences were reported in the honey bee worker venom gland [14]. These results bolster our view that CBPs are probably expressed by and play important roles in insect venom glands.

*Pteromalus puparum* (Hymenoptera: Pteromalidae) is a pupal-stage endoparasitoid of the small white cabbage butterfly, *Pieris rapae* (Lepidoptera: Pieridae), a serious pest of cruciferous crops. Venom is the sole *P. puparum* parasitic factor, responsible for manipulating host immunity and physiology [15]. *P. puparum* venom impairs host immune responses by suppressing host hemocytic defense reactions, including hemocyte spreading [16] and encapsulation [15,17] in the parasitized host. These effects on host hemocytes reduce host phenoloxidase activity [18], and change the total number and shape of host hemocytes [19]. *P. puparum* venom also manipulates host development through endocrine disruptions [20]. Several bioactive proteins from *P. puparum* venom have been characterized, including Vn11 [21], calreticulin [17], acid phosphatase [22], alkaline phosphatase [23], and an odorant binding protein [24]. Our analysis of the *P. puparum* venom apparatus transcriptome and venom proteome [25] identified a partial cDNA sequence encoding a chitin-binding-like protein (PpCBP) *in silico*. Here we report our confirmation of a PpCBP and report new information based on molecular and biochemical characterization of the protein. Based on this evidence, it is still unclear to estimate whether PpCBP is a toxin or non-toxin peptide. This is able to give us a new viewpoint that a gene expression product, the level of which is up-regulated in the parasitoid venom gland, may have no toxicological function to its host, and it was identified using transcriptomics-based approach.

## 2. Results

### 2.1. Full-Length PpCBP cDNA Cloning and Sequence Analysis

**Figure 1:**
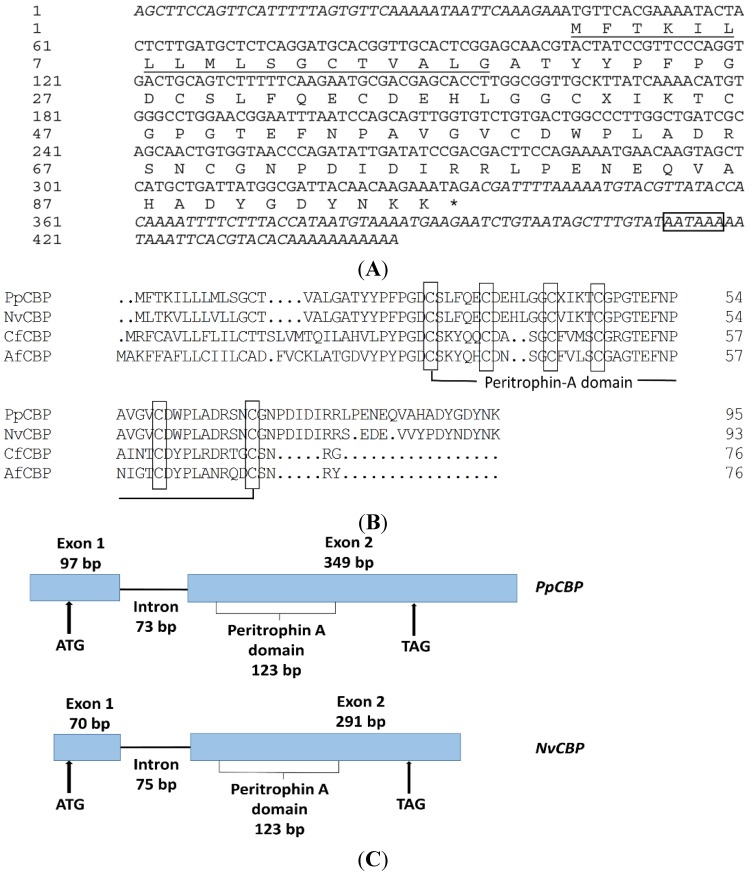
Sequence analysis of *PpCBP* in *Pteromalus puparum*. (**A**) The cDNA and deduced amino acid sequences of the *PpCBP*. The predicted signal peptide is underlined. Asterisk denotes a stop codon. The polyadenylation signal (AATAAA) is boxed; (**B**) Alignment of deduced amino acid sequences of PpCBP and three other insect species. The alignment was performed using the Clustalx program. NvCBP (*Nasonia vitripennis*), accession No. NP_001164343. CfCBP (*Camponotus floridanus*), accession No. EFN70974. AfCBP (*Apis florea*), accession No. XP_003694671. Cysteine residues in the mature proteins are boxed; (**C**) Schematic diagram of the *PpCBP* and *NvCBP* gene structure. Black boxes represent the two exons and the solid line represents the intron. Up arrows indicate positions of a start codon (ATG) and a stop codon (TAG). Numbers indicate the nucleotide length for the exons, peritrophin-A domain, and intron.

**Figure 2:**
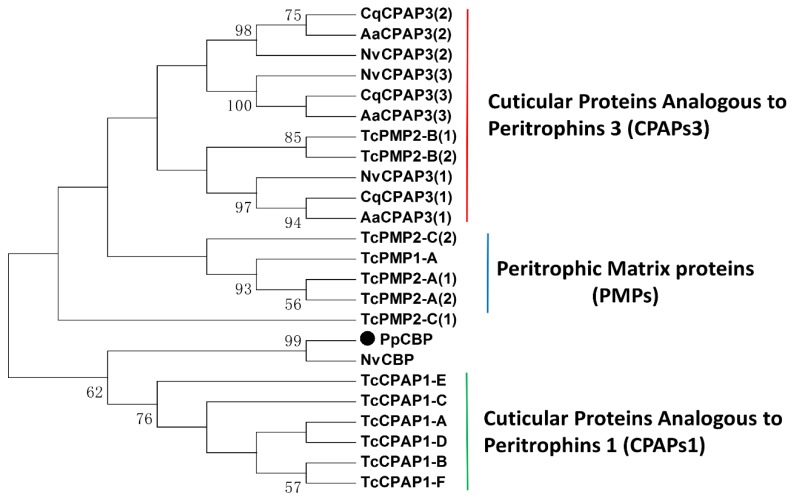
Phylogenetic analysis of the amino acid sequence of chitin binding domains within the cuticle proteins and peritrophic matrix proteins from the indicated insect species. The large dot highlights the PpCBP. The phylogenetic tree was constructed by using the maximum-likelihood method with a complete deletion of gaps. Numbers indicate bootstrap support values (%) based on 1000 replicates. The accession number of the orthologs from each species is listed in Table S1. Nv, *Nasonia vitripennis*; Tc, *Tribolium castaneum*; Cq, *Culex quinquefasciatus*; Aa, *Aedes aegypti*.

A full-length *PpCBP* cDNA was cloned using reverse transcription polymerase chain reaction (RT-PCR), as well as 5′- and 3′ rapid amplification of cDNA ends (RACE). The *PpCBP* cDNA contains a 42 bp 5′ untranslated region (UTR), 113 bp 3′ UTR, and a 291 bp open reading frame (ORF), encoding a protein of 96 amino acid residues (Figure 1A) including a secretory signal peptide (1–18aa) and a peritrophin-A domain (Figure 1B). Multiple alignment analysis showed that the consensus sequence of the peritrophin-A domain appears to be CX_5_CX_4-6_CX_4_CX_12_CX_9_C (where X is any amino acid except cysteine). Based on our genome database [26], we determined the *PpCBP* gene structure, which is similar to that of *NvCBP*, includes two exons and one intron (Figure 1C). The mature PpCBP has a predicted molecular mass of 8.59 kDa and a theoretical isoelectic point of 4.26. To establish the evolutionary relationships between PpCBP and other insect CBPs, a phylogenetic tree was constructed based on the amino acid sequences of the CBDs (Figure 2). Phylogenetic analysis of 24 CBDs from five insect species revealed the PpCBP CBD clustered with the CBD of NvCBP. The CBDs have been placed into three major groups, including cuticular proteins analogous to peritrophins 1 (CPAPs1), cuticular proteins analogous to peritrophins 3 (CPAPs3), and peritrophic matrix proteins (PMPs). PpCBP is clustered into the CPAPs1 type.

**Table 1:** Primers used in this study.

Primer Name	Nucleotide Sequence(5′–3′)
*PpCBP*-5′RACE-outer	ATTAAATTCCGTTCCAGGCCCACAT
*PpCBP*-5′RACE-inner	CGATCAGCCAAGGGCCAGTCACAGA
*PpCBP*-3′RACE-outer	CGATCAGCCAAGGGCCAGTCACAGA
*PpCBP*-3′RACE-inner	ACGAGCACCTTGGCGGTTGCGTTAT
*PpCBP*-SP *	CGCGGATCCGCAACGTACTATCCGTTCCCAG
*PpCBP*-AP *	CCGCTCGAGCTATTTCTTGTTGTAATCGCCATA
q*PpCBP*-SP	ACTATCCGTTCCCAGGTGAC
q*PpCBP*-AP	CATTTTCTGGAAGTCGTC GGA
q*Pp18s*-SP	CGAGCGATGAACCGACAG
q*Pp18S*-AP	CGGGGAGGTAGTGACGAA

* Restriction sites are underlined.

### 2.2. Tissues and Temporal Expression of PpCBP

**Figure 3:**
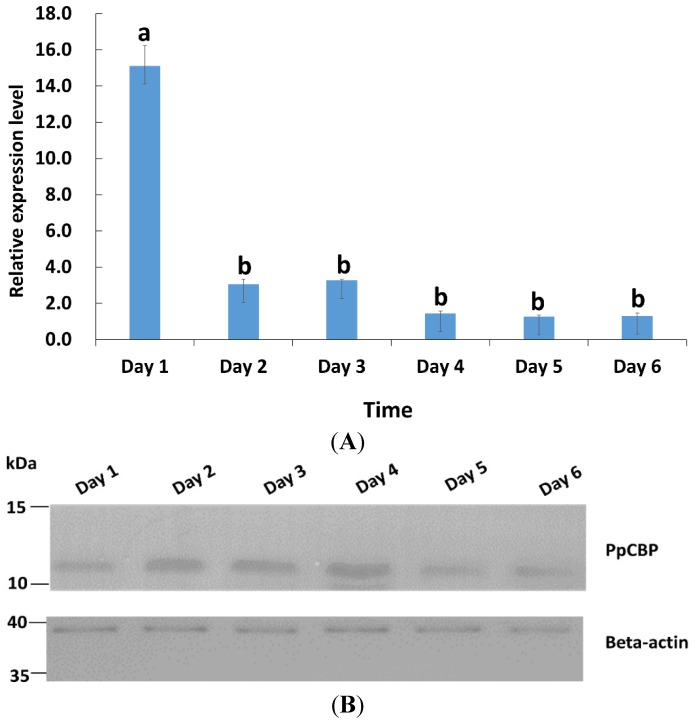
Relative expression of the *PpCBP* mRNA and its protein in adults aged day 1 to 6. (**A**) qPCR analysis; *18s rRNA* gene was used as a reference; each histogram bar represents the mean ± 1 SEM (*n* = 3) of transcript levels, and the bars annotated with the same letter are not significantly different (Tukey-HSD test, *p* < 0.05); (**B**) Western blot analysis, using β-actin as a loading control.

**Figure 4:**
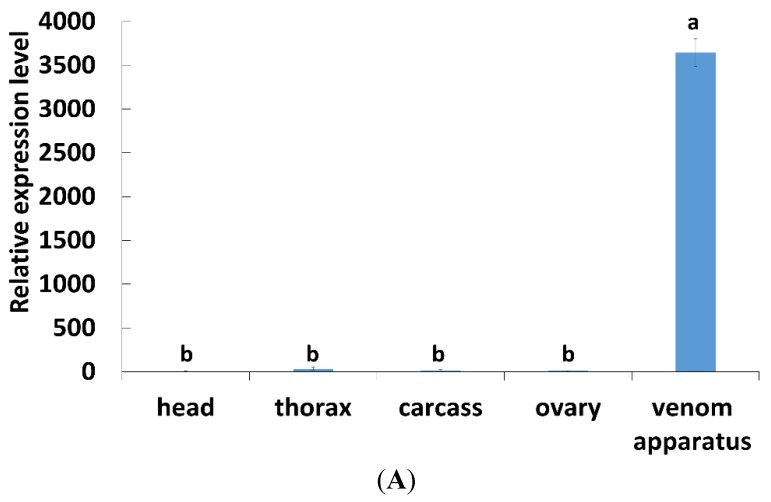

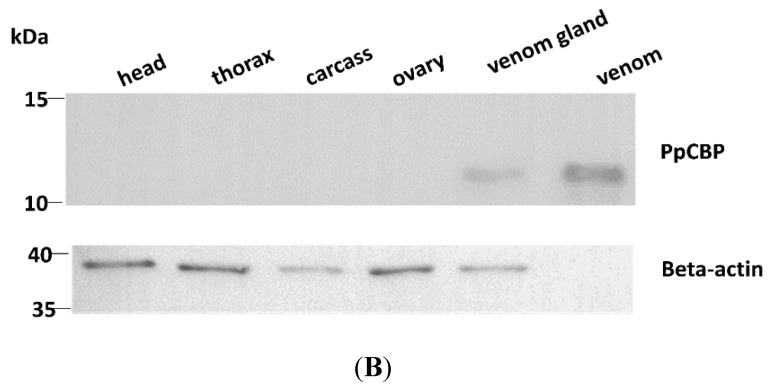
Tissue expression profiles of *PpCBP*. (**A**) qPCR analysis of the indicated tissues using *18s rRNA* as reference gene; each histogram bar represents the mean ± 1 SEM (*n* = 3) of transcript levels. Bars annotated with the same letter are not significantly different (Tukey-HSD test, *p* < 0.05); (**B**) Western blot analysis of the indicated tissues; β-actin was used as internal control.

The temporal expression profiles of *PpCBP* in venom glands from day 1 to day 6 in post-eclosion adults were studied by using real-time quantitative PCR (qPCR). The expression profiles of *PpCBP* were highest at day 1, then declined by about three- to five-fold (*p* < 0.05) by day 2, and remained very low for the following four days (Figure 3A). Western blot analysis (Figure 3B) shows that the PpCBP protein was present during each day of the experiment in the venom apparatus. The *PpCBP* expression pattern among tissues, including head, thorax, ovary, venom apparatus, and abdomen carcass, was determined. The *PpCBP* transcript was highly expressed in the venom apparatus (*p* < 0.05), and virtually undetectable in the other tissues (Figure 4A). Western blot analysis shows that PpCBP protein was present in the venom and venom gland, but not in other tissues (Figure 4B), consistent with our qPCR result.

**Figure 5:**
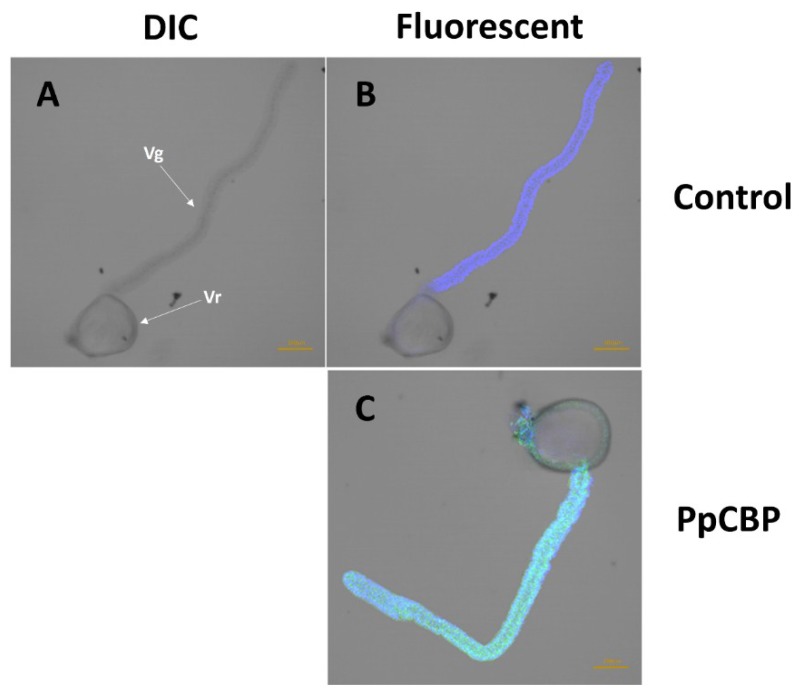
Immunohistochemical location of PpCBP in venom apparatus. (**A,B**) Control tissues incubated with only the secondary antibody; (**C**) Venom apparatus was incubated with anti-PpCBP antibody and FITC-conjugated second antibody. Nuclei of the venom apparatus stained blue with DAPI. (**A**) differential interference contrast (DIC) micrographs; (**B,C**) fluorescent micrographs. Vr, Venom reservoir; Vg, Venom gland.

We used an anti-PpCBP antibody and fluorescein isothiocyanate (FITC)-conjugated second antibody to visualize the expression and the location of PpCBP in the venom apparatus. We prepared a differential interference contrast (DIC) microphotograph to illustrate the anatomy of the venom apparatus, with a venom gland and venom reservoir identified (Figure 5A). The venom apparatus was mounted with 4′-6-diamidino-2-phenylindole (DAPI) to stain nuclei in the venom gland cells blue (Figure 5B). Figure 5C shows heavy PpCBP staining in the venom gland, with very little staining in the venom reservoir.

### 2.3. Recombinant PpCBP Expression Products and Cellulose- and Chitin-Binding Capacities

**Figure 6:**
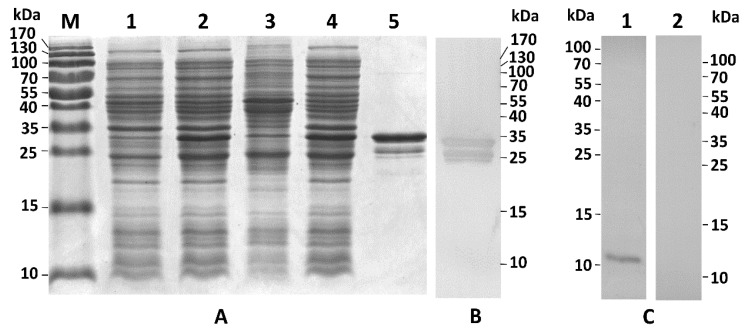
Recombinant PpCBP expression and specificity detection of the antiserum. (**A**) Prokaryotic expression and enrichment of recombinant PpCBP (rPpCBP); (**B**) Western blot analysis of enriched rPpCBP. Proteins were separated on 15% SDS-PAGE gel and stained with commassie brilliant R250. Lane M: marker, lane 1: uninduced *E. coli* BL21 (DE3) cells containing pGEX-4T-3-*PpCBP*; lane 2: induced BL21 (DE3) cells; lane 3: pellet of the induced bacterial lysate; lane 4: supernatant of the induced BL21 (DE3) cells; lane 5: enriched rPpCBP; (**B**) Western blot of enriched rPpCBP. The rPpCBP was analyzed by western blot using anti-GST antibody; (**C**) Detection specificity of the rPpCBP antibody. Total proteins of venom apparatus of *P. puparum* were separated on 15% SDS-PAGE gel, and analyzed by Western blot using rPpCBP antiserum (Lane 1) and pre-immune rabbit serum (Lane 2).

**Figure 7:**
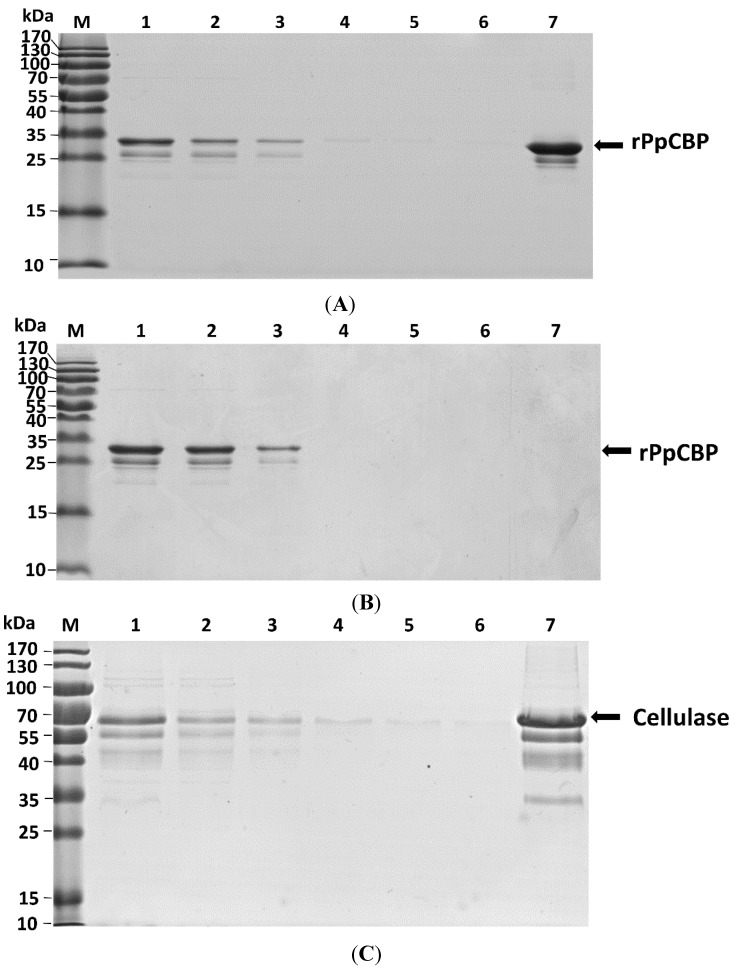
Recombinant PpCBP binds chitin, but not cellulose. (**A**) rPpCBP binds chitin. Lane M, protein molecular weight markers; lane 1, rPpCBP; lane 2, flow through fraction; lanes 3–6 consecutive washes 1–4; lane 7, proteins eluted from the column in the presence of 8 M urea; (**B**) rPpCBP does not bind cellulose. Lane identities just listed; (**C**) Cellulase binds cellulose. Lane identities listed for (**A**) except lane 1 was cellulase. The same volume for each fraction was loaded onto the gel except that the proteins in lane 7 were concentrated 10-fold.

The recombinant PpCBP (rPpCBP) was expressed in *Escherichia coli*. Analysis of expression products on 15% sodium dodecyl sulphate polyacrylamide gel electrophoresis (SDS-PAGE) revealed the presence of rPpCBP with the expected size of the fusion protein of 34.7 kDa in the supernatant of *E. coli* lysates. Figure 6 panel A, lane 1 shows a small band in the appropriate size range that may include rPpCBP as well as other proteins in the *E. coli* lysates that had not been induced with isopropyl β-d-1-thiogalactopyranoside (IPTG). A larger band in lane 2 indicates expression of the rPpCBP in IPTG-induced bacteria. Lanes 3 and 4 indicate the recombinant protein occurs in the supernatant rather than pellet fraction of the *E. coli* lysates. Lane 5 shows the enriched protein, which is confirmed by Western blot with anti-glutathione *S*-transferase (GST) antibody (Figure 6, Panel B). Panel C, lane 1 shows the anti-rPpCBP reacted with a single protein, indicating the specificity of the antibody. Lane 2 shows the antibody did not react with any protein present in the pre-immune serum. The chitin-binding ability was confirmed by the chitin-binding assay. Because the GST tag alone did not bind to cellulose and chitin [27], the rPpCBP was directly used to perform the binding assay. As shown in Figure 7A, the chitin panel, although the flow-through fraction contained rPpCBP, the fusion protein was eluted with 8 M urea in the chitin-binding assay. In Figure 7B, most of the rPpCBP was in the flow-through fractions and no protein was eluted with 8 M urea in the cellulose-binding assay. Panel 7C is a positive control, showing that cellulase binds to cellulose. We infer that rPpCBP specifically bound to chitin but not to cellulose.

## 3. Discussion

In this study, we identified and sequenced a cDNA likely encoding a CBP from the venom gland of *P. puparum*, designated *PpCBP*. Multiple alignment results showed that the structure of PpCBP was similar to NvCBP, which has only one chitin binding peritrophin-A domain. The deduced amino acid sequence of CBD in PpCBP is almost identical to that of NvCBP. Additionally, the phylogenetic tree placed the CBD of PpCBP with the CBD of NvCBP as a group. *P. puparum* and *N. vitripennis* are both Pteromalidae. However, the parasitic types of *P. puparum* and *N. vitripennis* are different. The structures of the two proteins revealed that they may have same functions. Chitin binding peritrophin-A domains appear in most insect CBDs as CX_11-30_CX_5-6_CX_9-24_CX_12-17_CX_6-12_C, where X is any amino acid except cysteine [28]. The consensus of conserved cysteines in PpCBP appears to be CX_5_CX_4-6_CX_4_CX_12_CX_9_C, which differs compared to the predicted chitin-binding sequence in other insects, because PpCBP has two shortened stretches between the first and second and the third and fourth cysteine residues. Our interpretation is these differences represent profound changes in insect CBPs that are supposed to have the same function. More data on a wider range of parasitoid venom proteins will be necessary to establish a link between the sequences of CBPs and their specific functions.

A chitin binding peritrophin-A domain is commonly present in insect PM proteins, insect cuticule proteins, chitin deacetylases, and chitinases [29]. PpCBP contains a signal peptide followed by a chitin binding peritrophin-A domain. The structure is similar to Ag-Aper14, a peritrophic matrix protein from *Anopheles gambiae* [30]. Ag-Aper14 is secreted from epithelial cells in *A. gambiae* after a blood meal. Ag-Aper14 was proposed to be a component of the peritrophic matrix that surrounds the ingested blood meal. Likewise, peritrophin-15, a type 2 peritrophic matrix protein with a similar structure to PpCBP, was identified from the old world screwworm fly, *Chrysomya bezziana*, and sheep blowfly, *Lucilia cuprina* [31]. Peritrophin-15 may act in capping the ends of individual chitin polymer chain, to protect them from degradation by exochitinase or to control the length of the polymerized chitin fibers. The *PpCBP* gene and the protein are highly expressed in the venom apparatus, but not in other tissues. We found that the PpCBP is located mainly in the cells of the venom gland, with some of the protein localized in the membrane of the venom reservoir by immunohistochemical detection. We had used the antibody to test whether the PpCBP is injected by the parasitoids. The Western blot signal is negative. However, the negative Western blot is not sufficient evidence to document the point. These results indicate to us that the PpCBP was synthesized and secreted by the venom gland, where it would serve functions in the venom apparatus or have functions in the host since PpCBP may be injected into the host hemocoel along with the parasitoid wasp eggs.

Our view is supported by the results of other experiments reported here. Transcripts encoding the protein are highly expressed in day 1 adults, possibly to complete construction of the venom apparatus, or perhaps for synthesis and accumulation of the proteins in the venom reservoir; the considerably lower expression in the following days is likely to serve in maintenance of the apparatus, or sustain the amount of the proteins for successful parasitization. We infer that the protein likely serves one or more biological functions within the venom apparatus or in the host hemocoel.

CBPs from various sources exhibit binding specificities. The bacterial CBP21 from *Serratia marcescens*, a non-catalytic CBP necessary for chitin catabolism [3], for example, binds far more squid chitin compared to other insoluble polysaccharides, including colloidal chitin and chitosan [32]. Human macrophages express a chitinase that includes a CBD. Ujita *et al.* [33] created a recombinant CBD (rCBD) to investigate its binding activity. They found that the rCBD binds specifically with hyaluronan and *N*-linked oligosaccharides associated with glycoproteins, but not to other carbohydrates. The authors suggested the CBD acts in tissue remodeling through binding to specific polysaccharides or matrix glycoproteins. Hardt and Laine [34] reported that the basis of chitin-binding specificity in the CBD of *Bacillus circulans* chitinase A1 lies in the active site amino acids within the CBDs, showing that creating amino acid substitutions in the active sites altered specificity for several substrates. It follows that binding specificity among CBPs and CBDs is an evolved feature of these proteins.

Our binding assay showed that the fusion proteins can bind to chitin *in vitro*, but not to cellulose, indicating to us that the PpCBP can interact with chitin. Shen and Jacobs-Lorena [35] identified a mosquito peritrophic matrix protein, *Anopheles gambiae* adult peritrophin 1 (Ag-Aper1), with two tandem CBDs. The authors inferred from the double CBDs that this protein acts as a molecular connector, tying peritrophic matrix chitin fibrils into a three-dimensional (3D) network. In their work on the evolution of CBDs, Shen and Jacobs-Lorena [6] noted that plant and invertebrate CBDs share structural features and chitin-binding mechanisms, arguing these common features result from convergent evolution. For Ag-aper1, the authors speculate that the spacing of the aromatic residues and possibly other residues within the CBDs are important features that define the binding specificity toward either chitin or cellulose.

The venom reservoirs of parasitoids are chitin-lined [36], and we speculate that PpCBP binds chitin in venom reservoirs, where it serves as a structural component of venom reservoirs. Also, chitin is a major component of the insect cuticle [37]. During the parasitization process, the parasitoid can injure the host exoskeleton, which may lead to microbial infections. We speculate that PpCBP functions to improve wound healing by accelerating the biosynthesis of chitin around the injury site. More experimental evidence, outside the scope of this work, will be necessary to move this speculation onto firmer ground. One the other hand, the evidence present in this manuscript also probably provides an example of a non-toxin peptide highly expressed in the venom gland compared to other tissues. Beyond the functions of CBP, this has implications for transcriptomics-based venom studies, which often use up-regulation as evidence for a role in secreted venom, which may have actively biological functions.

## 4. Experimental Section

### 4.1. Insects and Sample Preparation

The *P. puparum* colony and *P. rapae* pupae were collected from a suburb in Hangzhou, Zhejiang, China, and maintained as described by Cai *et al.* [15], at 25 °C, 80% relative humidity, and a 16L:8D photoperiod. After emergence, *P. puparum* adults were collected and held in glass vials. They were maintained on a 20% honey solution.

To investigate *PpCBP* tissue and developmental expression pattern, the heads, thoraxes, ovaries, venom apparatuses, and carcasses of day 3 adults and the venom apparatuses from day 1 to 6 wasps were dissected, and stored at −80 °C until subsequent processing. One hundred tissues were collected for each replicate, with three independent biological replicates.

### 4.2. Full-Length cDNAs Cloning

Total RNA was extracted from 100 venom glands using TRIzol (Invitrogen, Carlsbad, CA, USA) reagent following the manufacturer’s instructions. Then 5′ RACE and 3′ RACE ready cDNA was prepared using a 5′/3′ RACE Kit (Takara, Shiga, Japan) according to the manufacturer’s instructions. Based on the partial sequence of *PpCBP* obtained from our transcriptome, 3′ and 5′ gene-specific RACE primers were designed using Primer Premier 5 (PREMIER Bio-soft Int., Palo Alto, CA, USA). The 5′ and 3′ RACE reactions were performed by two semi-nested PCR reactions using the primers listed in Table 1. The 3′ and 5′ RACE PCR reactions were performed with LA Taq DNA polymerase (Takara). The PCR program includes 94 °C for 3 min, followed by 35 cycles of 94 °C for 30 s, 72 °C for 30 s, 55 °C for 30 s, followed by 68 °C for 7 min. The PCR products were analyzed on a 1.5% agarose gel. The target fragment was excised, and DNA was purified with AxyPrep DNA gel extraction kit (Axygen Scientific, Inc., Union City, CA, USA). The purified PCR products were directly cloned into a pGEM-T easy vector (Promega, Madison, WI, USA) and transformed into Trans-T1 competent *E. coli* cells (Transgen, Beijing, China). The positive clones were sequenced by Shanghai Biosune Sequencing Co. (Shanghai, China).

### 4.3. Sequence Analysis

DNASTAR SeqMan software (Lasergene, Madison, WI, USA) was used to assemble the cDNA sequence. The DNASTAR EditSeq program (Lasergene) was used to predict the isoelectric point (pI) and molecular weight (mw) of the deduced protein. Open Reading Frame Finder [38] was used to find the ORFs. The open access software AUGUSTUS version 2.4 [39,40] was used to predict the genomic structure with default settings. Basic Local Alignment Search Tool (BLAST) [41] was used to identify the sequence homolog. InterProScan 5 [42] was used to predict the conserved domains. The signalP4.0 [43] program was used to predict the secretory signal peptide. The ClustalX program [44] was used for sequence alignment. A phylogenetic tree for the PpCBP CBD with CBDs in other species was constructed with MEGA 5.10, using the maximum-likelihood method [45,46].

### 4.4. Recombinant Expression, Protein Purification, and Antiserum Preparation

The PpCBP N-terminal signal peptide was omitted. The mature protein fragment was amplified from venom gland cDNA with *PpCBP-SP* and *PpCBP-AP* primers (Table 1). The PCR reaction was carried out using a KOD plus neo kit (Toyobo, Osaka, Japan), according to the manufacturer’s instructions. The PCR conditions were: 98 °C for 3 min, followed by 45 cycles of 98 °C for 10 s, 68 °C for 30 s, followed by 68 °C for 7 min. The purified PCR products were digested with *BamH* I and *Xho* I, and subcloned into the prokaryotic expression plasmid pGEX-4T-3 (GE Healthcare, Piscataway, NJ, USA), generating the recombinant pGEX-4T-3-*PpCBP* plasmid.

For expression of the recombinant protein, the recombinant plasmid was transformed into competent *Escherichia coli* BL21 (DE3) cells (Transgen). The transformant was grown in ampicillin containing LB medium at 37 °C, 200 rpm, until the absorbance at 600 nm reached approximately 0.8. The cells were induced by 0.1 mM IPTG for 12 h at 12 °C. Cells were harvested by centrifugation (12,000× *g* for 1 min), suspended in BugBuster master mix (Novagen, Darmstadt, Germany), and lysed by shaking at room temperature for 20 min. The lysate was centrifuged at 12,000× *g* for 20 min at 4 °C, and then the supernatant and precipitate were examined by SDS-PAGE to determine whether the rPpCBP was present in the soluble or insoluble fractions.

The GST-tagged PpCBP was purified from the supernatant on affinity chromatography using a GST-Bind Resin Kit (Novagen). A column containing 1 mL of GST-bind resin was equilibrated with 5 volumes GST binding buffer (50 mM Tris, 200 mM NaCl, pH 8.0). The supernatant was loaded onto the column, and the column was washed with 10 volumes of GST washing buffer (50 mM Tris, 200 mM NaCl, pH 8.0) to remove non-specifically bound proteins. The protein was eluted with GST elution buffer (50 mM Tris, 200 mM NaCl, 10 mM reduced GSH, pH 8.0). For chitin- and cellulose-binding assays, fractions containing the rPpCBP were pooled and dialyzed against phosphate buffered saline (PBS) to remove the glutathione. The rPpCBP was analyzed by Western blot with anti-GST antibody (MultiSciences Biotech Co., Ltd, Hangzhou, China). Protein concentrations were determined by the bicinchoninic acid (BCA) method, and purity was analyzed by SDS-PAGE. The enriched rPpCBP was submitted to Hangzhou HuaAn Biotechnology Company (Hangzhou, China) to produce polyclonal antiserum. All the animal experimental protocols were approved by the Ethics Committee for Animal Experiment of Zhejiang University (Certificate No.: SYXK[zhe]2009-0142). Briefly, a male New Zealand rabbit was immunized subcutaneously with a mixture of 150 μg rPpCBP in 0.5 mL saline with an equal volume of complete Freund’s adjuvant. Two booster injections (150 and 75 μg rPpCBP) were given in incomplete Freund’s adjuvant each week. The serum was obtained one week after the third immunization to determine the PpCBP antibody specificity. The last injections (50 μg rPpCBP) were performed in incomplete Freund’s adjuvant one week later, and the antiserum was collected through the heart after one week. Pre-immune rabbit serum was collected at the same time as the negative control, total proteins prepared from the *P. puparum* venom apparatus were used for determining the specificity of the antiserum.

### 4.5. qPCR

Total RNA was isolated from the wasp venom apparatus collected from day 1 to day 6 adults. Total RNA was isolated from heads, thoraxes, ovaries, venom apparatus, and abdomen carcasses of day 3 adults to determine tissue-specific expression. The RNA samples were treated with DNase I (Ambion, Austin, TX, USA) to remove the contaminating genomic DNA. The RNA samples were reverse-transcribed into first-strand cDNA using random hexamer primers and a first-strand cDNA synthesis kit (Toyobo). The *18S rRNA gene* served as the reference gene, whose expression profile was the most stable compared with other housekeeping genes [24]. The qPCR primers (Table 1) were designed by Primer3 program [47]. The qPCR analyses were performed with three biologically independent replicates using SYBR^®^ Premix Ex Taq II (Takara) on a CFX96™ Real-Time PCR Detection System (Bio-Rad, Hercules, CA, USA). The cycling conditions were: 3 min at 95 °C by 40 cycles of 95 °C for 5 s, 60 °C for 30 s. A melt curve analysis was performed to ensure specificity of the amplification. A relative quantitative method (2^−ΔΔCT^) was used to evaluate the quantitative variation [48].

### 4.6. Western Blot

The PpCBP expression levels were determined by Western blot analysis. Total proteins were extracted from specific tissues of day 3 adults, including heads, thoraxes, ovaries, venom apparatus, and abdomen carcasses. Venom apparatuses were isolated from days 1 to 6 adults. The venom reservoirs in the isolated venom apparatuses were torn directly using forceps on a chilled concave slide, containing ice-cold phosphate-isolation buffer 10 mM sodium phosphate (pH 8.0), 0.9% (*w*/*v*) NaCl, 15% (*w*/*v*) sucrose, 1 mM ethylene diamine tetraacetic acid, and 1 mM phenylmethylsulfonyl fluoride, as described previously [21]. The venom fluids released into the buffer were collected and stored. The cracked venom apparatuses were rinsed in the clean buffer and then homogenized in the buffer using a tissue lyzer homogenizer (Qiagen, Hilden, Germany); the homogenate was centrifuged 12,000× *g* for 20 min. The supernatant solution contained the total crude protein extract from the venom apparatus, which was not contaminated with other proteins. All samples were separated on a 15% SDS-PAGE gel, and transferred to polyvinylidene difluoride membranes. The membranes were incubated overnight at 4 °C in Tris-buffered saline tween-20 (TBST) containing 5% non-fat milk. The membranes were incubated with primary anti-body (anti-PpCBP rabbit polyclonal antibody, diluted 1:1000) for 2 h at room temperature. After washing four times with TBST, the membranes were incubated with secondary antibody (Horseradish peroxidase-labeled goat anti-rabbit IgG (HuaAn Biotechnology Company; diluted 1:10,000)) for 1 h at room temperature. After washing with TBST and Tris-buffered saline, the proteins were visualized using 3,3′,5,5′-tetramethylbenzidine stabilized substrate (Promega). We used the expression product of β-actin as the reference, whose expression profile was more stable than other housekeeping genes [24].

### 4.7. Immunohistochemistry

Immunohistochemical staining was performed following Wang *et al.* [24]. In brief, the venom apparatuses were fixed with 4% paraformaldehyde for 0.5 h at room temperature in a 1.5 mL Eppendorf tube (Eppendorf, Oldenburg, Germany), and the tissues were subsequently permeabilized in buffer, phosphate buffered saline Triton X-100 (PBST), for 1 h. The tissues were rinsed 3 × 15 min with PBST, and then incubated overnight at 4 °C in PBST containing 5% bovine serum albumin (BSA). After rinsing 3 × 15 min with PBST, the venom apparatus were incubated in primary antibody for 4 h at room temperature. After rinsing again 4 × 15 min with PBST, the tissues were incubated with secondary goat anti-rabbit antibody conjugated with FITC (diluted 1:100 in PBS containing 1% BSA) for 0.5 h at room temperature in the dark and then rinsed in PBST for 3 × 15 min. Control apparatus were incubated with only the secondary antibody. After the rinsing, DAPI, 1 µg/mL in PBS, staining was performed at room temperature for 10 min, followed by several rinses in PBST. All images were acquired on a Leica TCS SP confocal microscope (Leica, Wetzlar, Germany) with a 10× objective.

### 4.8. Chitin- and Cellulose-Binding Assay

The chitin- and cellulose-binding assays were performed as described by Rebers and Willis [27] with minor modification. Briefly, chitin beads (New England BioLabs, Beverly, MA, USA) and cellulose powder (Sigma-Aldrich, St. Louis, MO, USA) were equilibrated in binding buffer (0.5 M NaCl, 10 Mm Tris, pH 7.0, 1.05%Triton X-100). For rPpCBP, 0.75 mg protein samples were diluted in 1.5 mL binding buffer, supplemented with protease inhibitor cocktail (Sigma-Aldrich). The 1.5 mL protein solution (0.5 mg/mL) was mixed with the 1 mL of chitin beads or cellulose powder and incubated 4 h in a polypropylene column at room temperature with gentle shaking. After incubation, the flow-through fraction was collected, and the column washed with four 1.5 mL aliquots binding buffer. The proteins bound to the beads were eluted with 1.5 mL of buffer containing 8 M urea. Cellulase (Sangon Biotech, Shanghai, China) was used as positive control in the cellulose binding assay as just described.

### 4.9. Statistical Analysis

The statistical analyses of the qPCR data were performed using a one-way ANOVA analysis with the data processing program of Tang and Zhang [49] (Version 9.50). Means were compared using a Tukey-HSD test (*p* < 0.05).

## Supplementary Materials

**Table S1.** The accession numbers of the orthologs from indicated species.
