# Supplementary material for: A Venom Gland Extracellular Chitin-Binding-Like Protein from Pupal Endoparasitoid Wasps, Pteromalus Puparum, Selectively Binds Chitin
Source: Toxins (Basel). 2015 Nov 30;7(12):5098–113. doi: 10.3390/toxins7124867 (PMC4690117; doi:10.3390/toxins7124867)
Supplement: Supplementary file 1 [file toxins-07-04867-s001.pdf]

Supplementary Materials

Table S1. The accession number of the orthologs from indicated species.

Name	Species	Accession NO.
TcPMP1-A	*Tribolium castaneum*	GU128096
TcPMP2-A	*Tribolium castaneum*	GU128099
TcPMP2-B	*Tribolium castaneum*	GU128100
TcPMP2-C	*Tribolium castaneum*	GU128101
TcCPAP1-A	*Tribolium castaneum*	GU128083
TcCPAP1-B	*Tribolium castaneum*	GU128084
TcCPAP1-C	*Tribolium castaneum*	GU128085
TcCPAP1-D	*Tribolium castaneum*	GU128086
TcCPAP1-E	*Tribolium castaneum*	GU128087
TcCPAP1-F	*Tribolium castaneum*	GU128088
CqCPAP3	*Culex quinquefasciatus*	XP_001868521
AaCPAP3	*Aedes aegypti*	XP_001662442
NvCPAP3	*Nasonia vitripennis*	XP_001607911
NvCBP	*Nasonia vitripennis*	NP_001164343
